# The impact of physiotherapy led education on time taken to mobilise in low risk patients following hepatobilary surgery

**DOI:** 10.1186/2197-425X-3-S1-A558

**Published:** 2015-10-01

**Authors:** F Williams, J Weblin, DJ McWilliams

**Affiliations:** Physiotherapy, University Hospitals Birmingham, Birmingham, United Kingdom; University Hospitals Birmingham, Birmingham, United Kingdom

## Introduction

ERAS protocols promote early mobilisation reducing risk of post-operative complication [1, 2]. The Southampton Physiotherapy Post-Operative Screening Tool (SPPOST) was introduced onto specialist surgical wards, with physiotherapy withdrawn for low risk patients. An audit concluded that mobility in this patient group was safely led by nurses, with no patients re-referred with pulmonary or mobility compromise. However, patients were not meeting their specific targets of walking >30 m by day 3 in line with the ERAS protocols.

## Objectives

Assess the impact of Physiotherapy led education for patients and nursing staff on time to mobilise >30 m and length of stay (LOS) in hepatobilary surgical patients.

## Methods

Patients admitted to an acute NHS trust undergoing non-specific hepatobilary surgery and who scored < 10 on the SPPOST were included. Patients who spent ≥3 days on ICU and/or developed a medical complication post-surgery were excluded. Baseline data was collected between 12^th^ July and 13^th^ August 2014. Following this posters promoting the benefits of mobilisation in the form of distance markers were displayed on the ward to encourage patients to mobilise. Education sessions were delivered to nursing staff to reinforce this ethos with patients. Data was collected prospectively from 11^th^ November to 13^th^ December 2014. Primary outcome was time taken to mobilise >30 m, with a secondary outcome of total LOS in days.

## Analysis

Differences between the pre and post intervention groups for time to mobilise > 30 m were analysed using a t-test (alpha ≤ 0.05) and presented as mean ± SD. Length of stay data was not normally distributed and therefore presented as median (IQR).

## Results

A total of 42 patients were identified as low risk during the study period and included in the analysis (20 in the baseline group and 22 in the post intervention group). Post intervention, a significant reduction was observed for time taken to mobilise 30 m compared to baseline patients (2.25 ± 0.71 days vs 2.82 ± 1.19 days, p < 0.05), with no significant difference in LOS between groups.

## Conclusions

The introduction of physiotherapy led education for patients and nursing staff resulted in a significant reduction in time taken to mobilise 30 m compared to baseline. Whilst more patients achieved targets set out in ERAS protocols, there was no significant difference in LOS. A further review of the management of ERAS patients is needed to ensure quality across all processes within the pathway.
